# Diseases of the Bladder and Prostate

**Published:** 1892-05

**Authors:** 


Disease of the Bladder and Prostate. By Hal. C. Wyman, M. Sc., M. D., Professor of Surgery in the Michigan College of Medicine and Surgery, Detroit; Member of the American Medical Association, Michigan State Medical Society, Michigan Surgical and Pathological Society, Detroit Academy of Medicine; Surgeon Detroit Emergency Hospital, etc., etc. The Physician's Leisure Library. George S. Davis, Detroit, Mich. 1891. Price, cloth, 50 cents; paper, 25 cents.

The importance of a complete understanding of the pathological management of diseases of the bladder has only lately come to be appreciated by the great mass of physicians. It has only recently been shown that an uncured gonorrhea leaves in its wake a multitude of diseases and disturbances that entail a life of suffering, or else shorten what might otherwise be a long and pleasant existence. Any contribution leading to a better understanding of these diseases is welcomed by physicians who properly appreciate their importance. The little volume before us is a condensation of the principal points that are presented in the management of the several diseases of the bladder and prostate. We commend it to the careful study of the profession.

